# Multi-View Human Action Recognition Using Skeleton Based-FineKNN with Extraneous Frame Scrapping Technique

**DOI:** 10.3390/s23052745

**Published:** 2023-03-02

**Authors:** Najeeb ur Rehman Malik, Usman Ullah Sheikh, Syed Abdul Rahman Abu-Bakar, Asma Channa

**Affiliations:** 1 Faculty of Electrical Engineering, Universiti Teknologi Malaysia, Johor Bahru 81310, Malaysia; 2 Computer Science Department, University Politehnica of Bucharest, 060042 Bucharest, Romania; 3 Department of Information Engineering, Infrastructure and Sustainable Energy, Mediterranea University of Reggio Calabria, 89100 Reggio Calabria, Italy

**Keywords:** HAR, skeleton, OpenPose, ML, FineKNN, EFS

## Abstract

Human action recognition (HAR) is one of the most active research topics in the field of computer vision. Even though this area is well-researched, HAR algorithms such as 3D Convolution Neural Networks (CNN), Two-stream Networks, and CNN-LSTM (Long Short-Term Memory) suffer from highly complex models. These algorithms involve a huge number of weights adjustments during the training phase, and as a consequence, require high-end configuration machines for real-time HAR applications. Therefore, this paper presents an extraneous frame scrapping technique that employs 2D skeleton features with a Fine-KNN classifier-based HAR system to overcome the dimensionality problems.To illustrate the efficacy of our proposed method, two contemporary datasets i.e., Multi-Camera Action Dataset (MCAD) and INRIA Xmas Motion Acquisition Sequences (IXMAS) dataset was used in experiment. We used the OpenPose technique to extract the 2D information, The proposed method was compared with CNN-LSTM, and other State of the art methods. Results obtained confirm the potential of our technique. The proposed OpenPose-FineKNN with Extraneous Frame Scrapping Technique achieved an accuracy of 89.75% on MCAD dataset and 90.97% on IXMAS dataset better than existing technique.

## 1. Introduction

Generally, images and videos contain useful information that can be capitalized to recognize embedded activities, actions or events. Human action recognition (HAR) is a branch of computer vision that involves identifying human actions in various scenarios. Traditionally, this process involves detecting local interest points or regions across time and space [1]. Conspicuously to its growing needs, HAR has recently gained significant of interest among researchers from various disciplines. Some of the key applications of HAR are in the field of security, surveillance, video retrieval, entertainment, assisted living, and human-computer interaction [1,2,3]. To ensure an effective and reliable HAR system, the following essential concerns are commonly considered:

1. **High performance**—performance of the action recognition technique is what determines the success of the human action system in terms of recognising actions.
2. **Region of interest**—essential parts of the image or video sequences that can be extracted or selected for action recognition.
3. **Computation complexity**—the time consumed by the system or algorithm to recognize the action.

**Figure 1:**
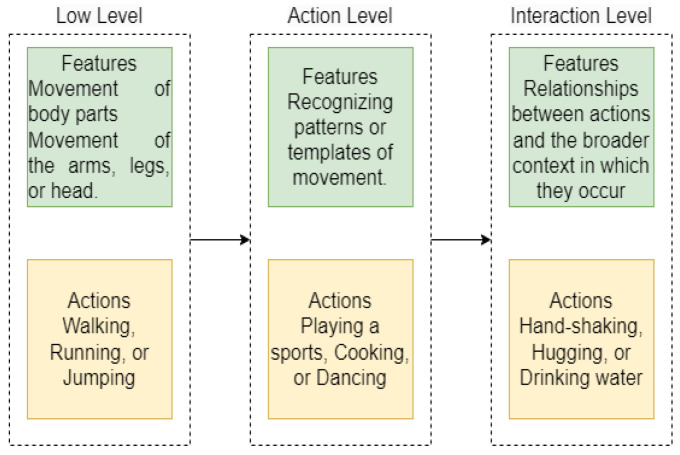
Intrinsic structure of human actions.

Typically, any actions performed by a human possess an intrinsic vertical structure consisting of multiple levels and are thus divided into three levels; low-level, action-level, and interactions-level as shown in Figure 1. The low level is also known as the atomic level i.e., hands waving, head shaking and facial expression. A composition of these atomic levels forms highly complex actions. The second level deals with the activities (actions) performed by a human i.e., walking, running and jogging. The third level deals with the actions that interact with other human(s) or object(s) [3] i.e., hand-shaking, hugging each other and drinking water.

The efficiency of any HAR system is based on its capability in extracting, modelling, and representing sailent, meaningful features [4]. Apparently, in the field of machine learning (ML) and computer vision, issues related to the extraction and representation of features are still being widely investigated [5]. Feature extraction is a process for converting arbitrary input data, such as images, videos, and text, into a set of features that describe patterns crucial during the recognition process [6]. In achieving the desired results, numerous feature extraction techniques take advantage of both the low and high level approaches. Cues obtained at these levels are further fused during the recognition process in obtaining qualitative results [7].

Meanwhile, for a real-time HAR system, model complexity is an important consideration for practical applications [8]. The overall complexity of the model greatly relies on the number of features. For instance, the higher the number of features, the greater the computational complexity [9]. one plausible approach to reduce the number of features is to use skeleton data as an alternative. Currently, the HAR system using skeleton data extracts the skeleton using OpenPose. For each detected body, a 2D skeleton with 25 joints is extracted. After that, the 2D skeletal features are transformed to RGB pictures. Finally, deep learning based classifiers are trained with the obtained RGB images for the HAR system [10].

Methods that exploit skeleton data have high performance in the field of HAR, but there are two major concerns when it comes to implementing this in real-world applications: Firstly, most CCTV cameras in the market are 2D RGB-based as they are inexpensive. on the other hand, the cost of installing or even replacing them with 3D cameras can be prohibitively expensive. Thus, enhancement based on the existing 2D sensors has become the best viable option. Secondly, 3D cameras involve additional depth information besides the normal RGB data which leads to an increase in storage requirement and computational time. Consequently, such requirements are unsuitable in the real-time image recognition problem. Moreover, 3D cameras typically have a limited working range which is unsuitable for surveillance purposes.

With the recent advancements in HAR techniques, several datasets based on multiple attributes such as single actor, multi-camera, open-view, and uncontrolled have been made available for use. These datasets are extensively utilized for comparing the accuracy of the newly proposed HAR systems with the previously developed HAR systems [11]. Some of the datasets found in previous studies by various researchers consist of more than one attribute, which make them more accurate. KTH [12] and Weizmann [13] datasets are based on only one attribute, which is a single actor, but they lack the multi-camera, open-view, and uncontrolled characteristics. Furthermore, IXMAS [14], i3D Post [15], and MuHAVI datasets [16] are based on two attributes, which are multi-camera and single actor. Hence, they are comparatively better than KTH and Weizmann. However, they also lack open-view and uncontrolled characteristics. Similarly, Hollywood, Olympic Sports, UCF11, and MSR datasets are also single attribute based datasets as they are only based on uncontrolled characteristic only. A better dataset based on three characteristics which includes multi-camera, single actor, and uncontrolled characteristics, is the MMA dataset. However, it lacks the open view attribute. Lastly, MCAD is the only dataset that contains all the said attributes, including multi-camera, single actor, open view, and uncontrolled, making it the most versatile dataset compared to all the other datasets. Therefore, this research utilizes the MCAD dataset for the development of skeleton based HAR system.

**Figure 2:**
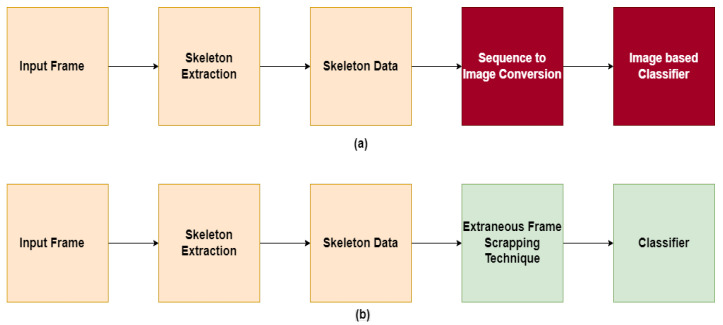
Skeleton-based approaches in HAR. (**a**) Existing method (**b**) Proposed method.

One unique feature about the MCAD dataset is that each action is always proceeded and ended with walking move. This may lead to confusion during the processing. This research proposes an Extraneous Frame Scrapping Technique for removing irrelevant walking frames so that the training phase could concentrate on the intended action. To sum up, this work offers the following contributions:

- A novel Extraneous Frame Scrapping Technique was proposed that addresses the problem of labeling walk as a different action.
- The methodology directly classifies skeleton data using a machine learning algorithm instead of converting it to RGB image, as discussed above and shown in Figure 2.

The remaining of this article is organized as follows. Section 2 discusses relevant literature, Section 3 describes the proposed method in depth. Section 4 covers the experimental setup. To demonstrate the efficiency of the proposed technique, the results and discussion are highlighted in Section 5, and finally followed by a brief conclusion in Section 6.

## 2. Related Work

Unsurprisingly, there have been a growing number of HAR approaches suggested in the related literature [17], and among these techniques that focused on feature extraction from depth data such as that of [18] are getting more attraction. The primary aim of such an approach is to assess the spatiotemporal depth sub-volume descriptors. According to Slama et al. [19], depth images are perceived as sequential features modeled temporally similar to subspaces positioned on the Grassmann manifold. In similar works, holistic descriptors such as the HON4D [20] and HOPC [21] were suggested, which rely on the orientations of normal surfaces in 4D, and are capable of representing the geometric attributes of a sequence of 3D points. Intensive 3D shape descriptor analysis has been performed in [22,23] order to determine the feasibility of 3D shape descriptors in object recognition or classification based on Kinect-like depth images. Wu et al. [11] developed a KNN classifier based on action descriptors such as the angular velocity and angular acceleration, as well as joint positions, to exploit human kinematic similarities in real-time. Munaro et al. [17] presented an autonomous framework that utilizes 3D motion flow for the real-time recognition of online human actions. This was accomplished with the application of the Microsoft Kinect sensor, which connects multi-point clouds to form an identifiable image by determining the RGB values of the current scenario on a grid-based description. After that, they classified the stored action using a KNN classifier [24]. In another development, Cao et al. [25] proposed a two-flow CNN architecture that leveraged on the OpenPose algorithm to extract the skeleton from RGB videos. Another work on skeleton based HAR system was proposed in [26]. An updated survey on handcrafted HAR can be found in [27]. Currently, the use of DL and the Kinect sensor in HAR systems is garnering a lot of interest due to the excellent outcomes they produce. The amount of computing required by such systems, on the other hand, is relatively large. Furthermore, when it comes to real-world scenarios, the RGB camera is the most typically available source of video input, which is incompatible with the models developed using Kinect data.

HAR interaction is a difficult task, as there are an assortment of human activities in day-to-day life. To handle this challenge, many DL models have been implemented. The accuracy of these DL models relies on the number of the training samples used. In the HAR tasks, a few datasets are openly accessible. These datasets incorporate a several activities like strolling, running, leaving a vehicle, waving, kicking, boxing, tossing, falling, twisting down, etc. Although a number of studies have used the DL architecture for HAR using 3D datasets as illustrated in Table 1, these datasets are unsuitable in currently installed systems, i.e., 2D camera-based surveillance system. Alternatively, several studies have focused on 2D datasets with enhanced accuracy. For instance, Baccouche and Mamalet [28] proposed an HAR System based on Convolutional Neural Networks (CNNs) and Recurrent Neural Networks (RNNs). They achieved an accuracy of 94.39% using the KTH Dataset. In another research by Ji et al. [29], instead of using CNN and RNN, they proposed s 3D-CNN and achieved a 90.02% accuracy using the KTH Dataset. Grushin et al. [30] used Long-Short-Term Memory (LSTM) architecture on the same KTH dataset and achieved an accuracy of 90.7%. Notwithstanding this relatively good accuracy, these methods are limited to only a single viewpoint dataset. In using a more challenging HMDB-51 dataset, Sun et al. [31] achieved an accuracy of 59.1%. In their work, a Factorized Spatio-Temporal CNN was employed. A similar attempt was made by Simonyan and Zisserman [32] using a two-stream CNN. They managed to achieve an accuracy of 59.4%. Wang and Qiao [29] succeeded in improving the accuracy performance of HAR to 65.9% using only the CNN architecture. Another deep CNN-based HAR system was proposed by Park et al. [33] based on the HMDB-51 dataset, achieving a relatively low accuracy of 54.9% accuracy.

In view of the limited performance of the methods discussed above, this work proposes a simpler strategy on a 2D dataset that can be used to cope with existing real-time systems. To avoid the time-consuming process of converting skeletal joints data to image sequences, and then training them using an image-based classifier that extracts the features again, the skeletal joints data be used directly for training and testing the HAR system. The discrepancy between prior approaches and the proposed approach can be best illustrated in Figure 2. We propose that a 1D feature vector be used as an input to the FineKNN classifier, which represents a simplification of the work that has been given.

## 3. Methodology

**Figure 3:**
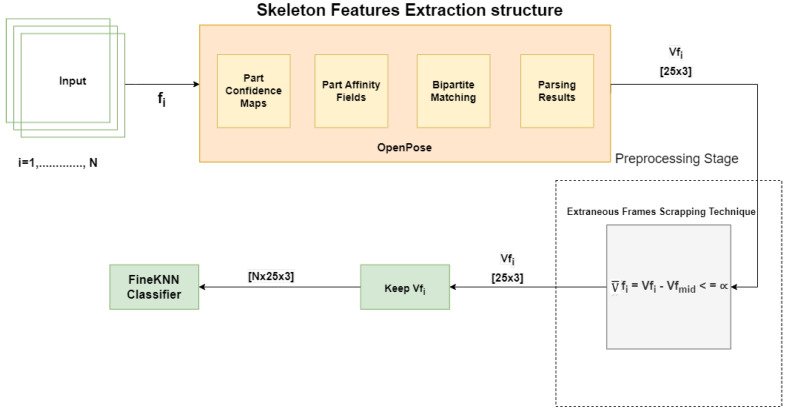
Proposed Methodology for HAR system based on skeleton data using EFS technique.

This section presents the proposed methodology for HAR in complex video sequences. The proposed design involves reading frames, extracting skeleton features, preprocessing and training the FineKNN classifier. The workflow of the proposed system is depicted in Figure 3. Initially, the video is loaded into the system. The system then read each frame while extracting the skeleton feature simultaneously. Next, the proposed Extraneous Frame Scrapping (EFS) Technique was applied to the extracted skeleton features. Finally, the output from EFS is fed into the FineKNN classifier for the classification process. The details of each step are provided below.

### 3.1. Extraneous Frame Scrapping Technique

In the MCAD dataset, most actions are proceeded and ended with the actor walking before performing the intended action. This makes walking frames redundant for HAR, and has potentional to contribute errors. To address such a problem, this work proposed an Extraneous Frame Scrapping Technique.

The proposed approach, although data-driven, can be generalized to any data set, irrespective of the frames under study. A middle frame is considered a reference frame, given as $Vf_{mid}$ as expressed in Equation (1). Next, a threshold α is chosen, which is calculated by taking the difference of different frames in video, i.e., the difference of action from an action frame, whereas the α value is calculated through experiments, as expressed in Equation (2). The frames resulting in >α are considered irrelevant, as those frames reduce the efficacy, resulting in inaccurate solutions. The threshold value α is calculated by taking the difference of various frames in the video, i.e., the difference of action from to action frame is less than α, whereas the difference of a particular action from walking is greater than α. Therefore, consideration of only a limited number of frames whose difference lies below the threshold value will lead to better action recognition and hence, increased accuracy.

(1) $$Vf_{mid}\equiv floor\left(\frac{N}{2}\right)$$

(2) $$Vf_{mid}-Vf_{i}\leq \alpha$$

however, α is ≤99, where $Vf_{mid}$ is the mid frame and $Vf_{i}$ is the current frame.

The threshold value α is a parameter that helps to determine whether two frames belong to the same action. The threshold is employed to calculate the difference between various frames in a video, and to decide whether to consider them as part of the same action. The value of α is typically set by comparing the difference between various actions. For example, the difference between two frames of the same action (e.g., walking) is typically less than α, whereas the difference between that action and a different action (e.g., waving) is typically greater than α. This allows the algorithm to distinguish between different actions based on the degree of similarity between the frames. The threshold value alpha is set by comparing the difference between the skeleton joints of the middle frame of an action, with the other frames in the sequence. The middle frame is chosen as a reference frame because it typically represents the optimal representation of the action, and it captures the essential elements of the action. Next, the difference between the skeleton joints of the middle frame and the other frames is calculated. By taking the difference from the skeleton joints of other frames from the sequence and mid frame, it was observed that value of alpha was greater than 99 when the frames were taken from different actions, while it was less than 99 when both frames belongs to same action.

As shown in Figure 3, the proposed technique first obtains the features extracted using OpenPose. Next, the mid-frame of each video is then compared to all other frames in the video, and their differences are calculated. If the difference is greater than the threshold value, the frame is removed, as such a frame is considered irrelevant in that particular action. Otherwise, the frame is stored as a useful frame, thus contributing to enhanced efficiency. We only collect the specific frames for a particular action that is intended for training purposes. It is worthy to mention that this data preprocessing technique is only required during the training phase, while during the testing phase, the EFS technique is not required.

### 3.2. Features Extraction Using OpenPose

**Figure 4:**
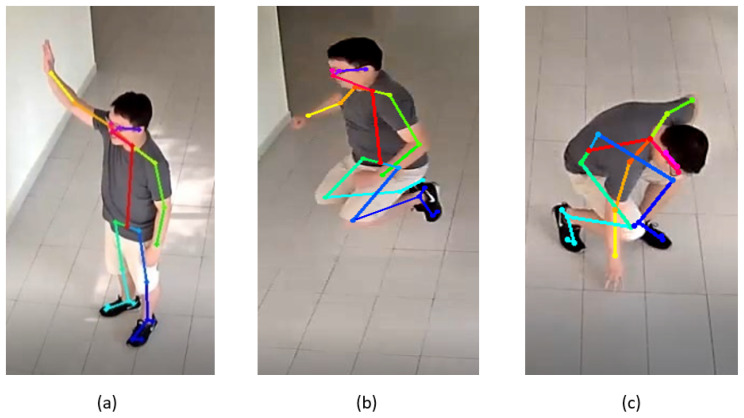
Skeleton detection of few actions from MCAD dataset. (**a**) Wave (**b**) Jump (**c**) SitDown.

For the skeleton extraction part, we used the existing OpenPose algorithm [19] since it has reliable performance in producing the skeleton features from the conventional 2D RGB images. Thus, there is no requirement to replace the existing systems with 3D imaging or depth sensor devices. The OpenPose algorithm is capable of detecting the 2D poses of several people in a given image. Realtime multi-person 2D pose estimation is a key component in enabling machines to have an understanding of people in images and videos. It uses a bottom-up approach to detect these 2D poses by capturing and locating body parts associated with people in the image. By default, OpenPose returns the position of the 25 joints (customizable) of each detected body as a vector of pairwise coordinates (x, y), along with the confidence score C, for each detected joint. The skeleton detection of several parts is shown in Figure 4.

### 3.3. Training Using FineKNN Classifier

The k-nearest neighbors (KNN) algorithm is a simple, supervised machine learning algorithm that can be employed to solve both classification and regression problems. The KNN classifier has been widely used in the fields of pattern classification and machine learning. For example, the KNN classifier has been applied for feature selection [47] and dimensionality reduction [48]. The conventional KNN classifier simply uses the K training samples that are nearest to the test sample to classify it. As pointed out by Weinberger et al. [49] the accuracy of KNN classification significantly depends on the metric used to compute the distances between different samples. KNN works by tracking down the distances between a query and every samplesin the dataset, choosing the predefined number models (K) nearest to the query, then, at that point, votes in favor of the most frequent label (on account of characterization), or averages the labels in the case of regression.

## 4. Experimental Setup

This study used the MCAD [50] and IXMAS [51] dataset, which are well-known for their uncontrolled and multi-view motions, to demonstrate the performance of the proposed technique. They include 14,298 action examples, performed by 20 individuals and recorded by five cameras [50]. There are 18 actions involved in this experiment, as mentioned in Table 2. Class one to nine belong to the single person action category, whereas class ten to eighteen belongs to interaction level actions. The dataset is divided into two parts; 80% data was used for training the model, while the remaining 20% was used for testing purposes.

For further evaluation of the proposed system, all experiments were performed on the IXMAS dataset. IXMAS has 12 action categories and 1800 action samples performed by 12 actors, and five cameras recorded them. There are 12 actions involved in this experiment, as mentioned in Table 3. The IXMAS dataset was also divided into 80% for training and 20% for testing.

**Figure 5:**
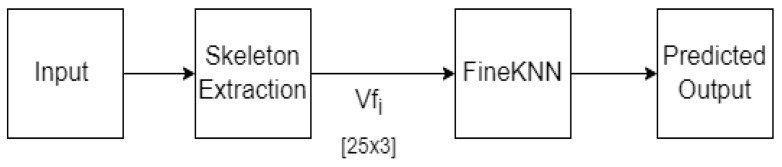
Testing phase of the proposed system.

For the proposed method from all the RGB videos in the database, the subject’s skeleton was first extracted using the OpenPose algorithm. We followed the process flow presented in Figure 5 for the testing phase, which is similar to the training phase. once trained, we can feed either the pre-recorded or the live video into the system as input. once the features were extracted, the proposed method would then classify the actions in the video. For analysis, we then compared the accuracy of these results against the actual given labels of the particular video.

The evaluation metrics used in this paper are a confusion matrix and Area Under the Curve (AUC). A confusion matrix usually allows performance visualization of a supervised algorithm. The positive and negative labels refer to the outcome of the classifier, while true/false shows the actual label as shown in Table 4.

The AUC of a classifier is a metric that assesses its ability to distinguish between distinct classes. This metric assesses how well the model distinguishes between the positive and negative categories. The higher the value, the better the performance of the system. If the value of AUC is 0, the performance of the classifier is poor. A value of 1, indicates that the performance of the classifier is optimal. The True Positive Rate (TPR), Specificity, and False Positive Rate (FPR) can be calculated as shown in Equations (3)–(5) respectively. To measure AUC, the ROC curve is first constructed by plotting the TPR against the FPR for a range of different classification thresholds. The TPR is calculated as the number of true positive predictions divided by the total number of positive instances, while the FPR is calculated as the number of false positive predictions divided by the total number of negative instances.

Once the ROC curve has been constructed, the AUC is calculated as the area under the curve. This can be done by numerically integrating the ROC curve, or by counting the number of points above and below the curve and calculating the fraction that are below the curve. AUC values range from 0.5 to 1, with a value of 1 indicating perfect classifier performance, and a value of 0.5 indicating random performance.

(3) $$TPR=\frac{TP}{TP+FN}$$

(4) $$Specificity=\frac{TN}{TN+FP}$$

(5) FPR=1−Specificity

## 5. Results and Discussions

To illustrate the efficacy of the proposed method, we demonstrated the performance of the proposed approach in several forms. First, we explored several KNN-based classifiers along with SVMs and Fine Tree classifiers. At the classification stage, we proposed the use of the FineKNN classifier. We implemented the different ML algorithms such as Medium KNN, Coarse KNN, Fine KNN, Cosine KNN, Weighted KNN, SVM, and Fine KNN. The Fine KNN outperformed every other algorithm in terms of accuracy. As shown in Table 5, Fine KNN achieves the highest performance. The results we achieved with the EFS technique demonstrate that we managed to obtain near 89.75% accuracy.

**Figure 6:**
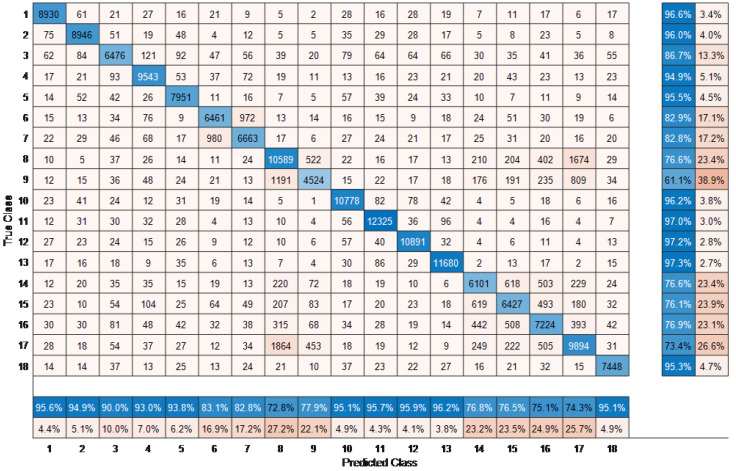
Confusion matrix without EFS technique.

Next, to demonstrate the efficacy of the proposed method with respect to each individual class, we implemented Fine KNN with and without the EFS technique. This experimentation was used to compare with our previous work, whereby no preprocessing stage was considered [22]. The confusion matrix for the MCAD dataset without the EFS technique is shown in Figure 6. The overall performance accuracy obtained was 86.99%. The last two columns show the average accuracy and average error per individual true class. Since no preprocessing stage was initiated, all frames, including the irrelevant frames were used. By accumulating only the useful frames (i.e., those that have lower than α difference from the reference mid frame $Vf_{mid}$), the performance accuracy increases by 3% to 89.75%. The proposed methods accuracy improves due to the proposed EFS technique, which eliminates the frames from the training data that does not belong to a particular action.

The receiver operating characteristic (ROC) curve was used to further demonstrate the performance of the suggested approach. This is a classification problem evaluation metric that compares the true positive rate (TPR) against the false positive rate (FPR) at various threshold values on a probability curve. The ROC curve is a representation of a classifier’s ability to differentiate across different discrete classes. The AUC values for all 18 actions are shown in Table 6. For example, Class 1 has an AUC of 0.97, indicating that the classifier can differentiate Class 1 from the other 17 classes with a 97% accuracy. We further tested our proposed system with the Extraneous Frame Scrapping Technique with the IXMAS dataset. Table 7 shows the AUC values for all the 12 action classes.

Finally, we compared our proposed method (both with and without EFS technique) with the state-of-the-art methods using overall accuracy, as shown in Table 8. Comparatively, our model improved by 32.9% accuracy from that achieved previously by Cuboid features [50], 25.3% from the Covariance matrices [52], 15.1% from the CNN-LSTM, 8.5% from the STIP features [50], 5.5% from the IDT [34] and 2.8% from the Conflux LSTM network [53]. These results confirmed the superior performance of our method.

For further validation with the state-of-the-art we executed our proposed method on the IXMAS dataset using overall accuracy, as shown in Table 9. Comparatively, our model improved by a 1.22% accuracy from that achieved previously by Shape Features [56], 10.42% from LBP [45], 7.94% from Motion Features [44], 5.17% from Shape features [57] and 0.06% from Shape Features(3D) [58]. These results confirmed that the proposed method is suitable for performing multi-view human action recognition.

We also performed complexity analysis between our proposed method and other existing methods by leveraging the size of the features dimension. Table 10 lists the comparison results with both the handcrafted and DL methods. The table clearly indicates that by far, the proposed method outperformed other existing methods in terms of feature dimensions. Our approach uses 75 features in total which is significantly small compared to other DL approaches.

## 6. Conclusions

Human Action Recognition has been a topic of active research in recent years. one of the primary goals of this research has been to address the complexity challenges involved in recognizing human actions from video data. This can be seen in the great effort made by previous works to simplify the process of action recognition. In the current study, we proposed a method for human action recognition that addresses the complexity issue by using a lower number of input features. The method starts by extracting 2D skeleton data from the 2D RGB data using the OpenPose technique. This 2D skeleton data provides a compact representation of human poses and reduces the complexity of the data that needs to be processed. The next step involves removing irrelevant information from the skeleton data using the Extraneous Frame Scrapping Technique. This further simplifies the data and reduces the complexity of the recognition process. Finally, the processed data was classified using a Fine-KNN classifier. The Fine-KNN classifier was chosen for its ability to perform classification based on a small number of features. Consequently, our proposed method significantly decreases the complexity of computation by reducing the feature dimensions. In this context, the proposed method was compared with other existing methods, and the results obtained confirm the potential of our proposed technique.

## Figures and Tables

**Table 1:** Review on techniques for Human Action Recognition.

Authors	Methods	Datasets	Accuracy (%)	Limitations
Baccouche and Mamalet 2011 [28]	CNN and RNN	KTH	94.39	Single viewpoint-based dataset
Ji et al., 2013 [29]	DCNN	KTH	90.02	Single viewpoint-based dataset
Grushin et al., 2013 [30]	LSTM	KTH	90.70	Single viewpoint-based dataset
Sun et al., 2015 [31]	Factorized spatio-temporal CNN	HMDB-51	59.10	Lower Accuracy
Simonyan and Zisserman, 2014 [32]	Two stream CNN	HMDB-51	59.40	Lower Accuracy
Wang and Schmid, 2013 [34]	Dense trajectory	HMDB-51	57.2	Lower Accuracy
Peng et al., 2014 [35]	Fisher vector and Stacked Fisher Vector	HMDB-51	66.79	Lower Accuracy
Wang and Qiao, 2015 [36]	CNN	HMDB-51	65.90	Lower Accuracy
Ahsan et al., 2018 [37]	GAN	UCF101 HMDB51	47.20 14.40	Lower Accuracy
Shi et al., 2019 [38]	AGCNLSTM	NTU-RGBD (CV) NTU-RGBD (CS)	95.00 89.20	Depth data
Li et al., 2019 [39]	CNN	UTD-MHAD NTU-RGBD (CS)	88.10 76.20	Depth data
Zhou et al., 2018 [40]	Two-stream MiCT	HMDB51	70.50	Lower Accuracy
Feichtenhofer et al., 2016 [41]	CNN	HMDB51	65.20	Lower Accuracy
Akilan et al., 2017 [42]	ConvNets	CIFAR100	75.87	Lower Accuracy
Fogia et al., 2014 [43]	DBN	MHAD MIVIA	85.80 84.70	Depth Data
Chun and Lee, 2016 [44]	Motion Features	IXMAS	83.03	Sensitivity to viewpoint changes
Baumann et al., 2016 [45]	LBP	IXMAS	80.55	Sensitivity to viewpoint changes
Lin et al., 2009 [46]	Shape-motion	IXMAS	88.89	Sensitivity to viewpoint changes

**Table 2:** List of actions from MCAD Dataset.

Class	Action
1	Point
2	Wave
3	Jump
4	Crouch
5	Sneeze
6	SitDown
7	StandUp
8	Walk
9	PersonRun
10	CellToEar
11	UseCellPhone
12	DrinkingWater
13	TakePicture
14	ObjectGet
15	ObjectPut
16	ObjectLeft
17	ObjectCarry
18	ObjectThrow

**Table 3:** List of actions from IXMAS Dataset.

Class	Action
1	check-watch
2	Cross-arms
3	Get-up
4	kick
5	Pick-up
6	Point
7	Punch
8	Scratch-head
9	Sit-Down
10	Turn-Around
11	Walk
12	Wave

**Table 4:** Confusion Matrix Example.

	True	False
Positive	True Positive	False Positive
Negative	True Negative	False Negative

**Table 5:** Classifiers testing on Skeleton Data.

Algorithm	Accuracy
Medium KNN	73.00%
Coarse KNN	48.00%
Cosine KNN	72.70%
Weighted KNN	82.20%
SVM	81.50%
Fine Tree	71.00%
**Fine KNN**	**89.75%**

**Table 6:** The area under the curve as per class on MCAD dataset with EFS technique.

Action	Class	AUC
1	Point	0.97
2	Wave	0.98
3	Jump	0.93
4	Crouch	0.96
5	Sneeze	0.96
6	SitDown	0.94
7	StandUp	0.91
8	Walk	0.89
9	PersonRun	0.82
10	CellToEar	0.97
11	UseCellPhone	0.98
12	Drinking Water	0.98
13	TakePicture	0.98
14	ObjectGet	0.91
15	ObjectPut	0.90
16	ObjectLeft	0.88
17	ObjectCarry	0.88
18	ObjectThrow	0.97

**Table 7:** The area under the curve as per class on IXMAS dataset with EFS technique.

Action	Class	AUC
1	Check-watch	1.0
2	Cross-arms	0.93
3	Get-up	0.94
4	Kick	0.95
5	Pick-up	0.91
6	Point	0.88
7	Punch	0.86
8	Scratch-head	0.91
9	Sit-down	0.94
10	Turn-around	0.82
11	Walk	0.84
12	Wave	0.83

**Table 8:** Multi-view HAR on MCAD dataset.

Algorithm	Accuracy
Cuboids [50]	56.8
Covariance matrices [52]	64.3
LSTM-CNN	74.6
STIP [50]	81.7
IDT [34]	84.2
Conflux LSTM network [53]	86.9
C3D + T-VLAD [54]	78.6
OpenPose+FineKNN (without EFS Technique) [55]	86.9
**OpenPose+FineKNN (with EFS Technique)**	**89.7**

**Table 9:** Multi-view HAR on IXMAS dataset.

Algorithm	Accuracy
LBP [45]	80.55
Motion Features [44]	83.03
H-VLBP [59]	84.5
T-VLAD [54]	84.8
Shape features [57]	85.80
MLDL [60]	89.6
Shape Features [56]	89.75
Shape Features (3D) [58]	90.91
**OpenPose+FineKNN (with EFS Technique)**	**90.97**

**Table 10:** Comparison of feature dimension.

Algorithm	Year	Method	Data Used	Feature Dimension
HON4D [20]	CVPR 2013	Handcrafted (global descriptor)	Depth	[17,880, 151,200]
HDG [21]	WACV 2014	Handcrafted (local + global descriptor)	Depth + skeleton	[1662, 1819]
P-LSTM [61]	CVPR 2016	Deep learning (LSTM)	Skeleton	No. of joints × 3×8
HPM + TM [62]	CVPR 2016	Deep learning (CNN)	Depth	4096
Clips + CNN + MTLN [63]	CVPR 2017	Deep learning (pre-trained VGG19, MTLN)	Skeleton	7168
RNN [64]	CVPR 2018	Deep learning (RNN)	Skeleton	512
ST-GCN [65]	AAAI 2018	Deep learning (Graph ConvNet)	Skeleton	256
**Proposed**	**2022**	**OpenPose + FineKNN**	**RGB**	**75**

## Data Availability

Not applicable.

## Abbreviations

The following abbreviations are used in this manuscript:

HAR	Human Action Recognition
EFS	Extraneous Frame Scrapping
HCI	Human Computer Interaction
STIP	Space Time Interest Point
BOW	bag-of-words
STVs	Space Time Volumes
PCA	Principal Component Analysis
CNN	Convolution neural network
LSTM	Long Short Term Memory
RNN	Recurrent Neural Network

## References

[1] Stottinger J., Hanbury A., Sebe N., Gevers T. (2012). Sparse color interest points for image retrieval and object categorization. IEEE Trans. Image Process..

[2] Sargano A., Angelov P., Habib Z. (2017). A comprehensive review on handcrafted and learning-based action representation approaches for human activity recognition. Appl. Sci..

[3] Zhang S., Wei Z., Nie J., Huang L., Wang S., Li Z. (2017). A review on human activity recognition using vision-based method. J. Healthc. Eng..

[4] Wu D., Zheng S., Zhang X., Yuan C., Cheng F., Zhao Y., Lin Y., Zhao Z., Jiang Y., Huang D. (2019). Deep learning-based methods for person re-identification: A comprehensive review. Neurocomputing.

[5] Bengio Y., Courville A., Vincent P. (2013). Representation learning: A review and new perspectives. IEEE Trans. Pattern Anal. Mach. Intell..

[6] Kumar G., Bhatia P. A detailed review of feature extraction in image processing systems. Proceedings of the 2014 Fourth International Conference on Advanced Computing & Communication Technologies.

[7] Liu Y., Gevers T., Li X. (2015). Color constancy by combining low-mid-high level image cues. Comput. Vis. Image Underst..

[8] Angerbauer S., Palmanshofer A., Selinger S., Kurz M. (2021). Comparing Human Activity Recognition Models Based on Complexity and Resource Usage. Appl. Sci..

[9] Wang L., Xu Y., Cheng J., Xia H., Yin J., Wu J. (2018). Human action recognition by learning spatio-temporal features with deep neural networks. IEEE Access.

[10] Aubry S., Laraba S., Tilmanne J., Dutoit T. (2019). Action recognition based on 2D skeletons extracted from RGB videos. MATEC Web Conf..

[11] Wu D., Sharma N., Blumenstein M. Recent advances in video-based human action recognition using deep learning: A review. Proceedings of the 2017 International Joint Conference on Neural Networks (IJCNN).

[12] Nada KTH Recognition of Human Actions. https://www.csc.kth.se/cvap/actions/.

[13] Gorelick L., Blank M., Shechtman E., Irani M., Basri R. (2007). Actions as Space-Time Shapes. Trans. Pattern Anal. Mach. Intell..

[14] IXMAS Dataset IXMAS Actions—New Views and Occlusions. https://www.epfl.ch/labs/cvlab/data/data-ixmas10/.

[15] University of Surrey and CERTH-ITI within the i3DPost Project i3DPost Multi-View Human Action Datasets. http://kahlan.eps.surrey.ac.uk/i3dpostction/.

[16] MLdta MuHAVi and MAS Human Actions. https://mldta.com/dataset/muhavi-and-mas-human-action/.

[17] Cippitelli E., Gasparrini S., Gambi E., Spinsante S. (2016). A human activity recognition system using skeleton data from RGBD sensors. Comput. Intell. Neurosci..

[18] Yang X., Tian Y. Super normal vector for activity recognition using depth sequences. Proceedings of the IEEE Conference on Computer Vision and Pattern Recognition.

[19] Slama R., Wannous H., Daoudi M. Grassmannian representation of motion depth for 3D human gesture and action recognition. Proceedings of the 2014 22nd International Conference on Pattern Recognition.

[20] Oreifej O., Liu Z. Hon4d: Histogram of oriented 4d normals for activity recognition from depth sequences. Proceedings of the IEEE Conference on Computer Vision and Pattern Recognition.

[21] Rahmani H., Mahmood A., Huynh D., Mian A. Real time action recognition using histograms of depth gradients and random decision forests. Proceedings of the IEEE Winter Conference on Applications of Computer Vision.

[22] As’ari M., Sheikh U., Supriyanto E. (2014). 3D shape descriptor for object recognition based on Kinect-like depth image. Image Vis. Comput..

[23] As’ari M.A., Sheikh U.U., Supriyanto E. (2014). XZ-shape histogram for human-object interaction activity recognition based on Kinect-like depth image. Wseas Trans. Signal Process..

[24] Khan M., Ware A., Karim M., Bahoo N., Khalid M. (2020). Skeleton based Human Action Recognition using a Structured-Tree Neural Network. Eur. J. Eng. Technol. Res..

[25] Cao Z., Hidalgo G., Simon T., Wei S., Sheikh Y. (2021). OpenPose: Realtime Multi-Person 2D Pose Estimation Using Part Affinity Fields. IEEE Trans. Pattern Anal. Mach. Intell..

[26] Malik N., Bakar S., Sheikh U. A Simplified Skeleton Joints Based Approach For Human Action Recognition. Proceedings of the 2021 IEEE International Conference on Signal and Image Processing Applications (ICSIPA).

[27] Abu-Bakar S. (2019). Advances in human action recognition: An updated survey. IET Image Process..

[28] Baccouche M., Mamalet F., Wolf C., Garcia C., Baskurt A. Sequential deep learning for human action recognition. Proceedings of the International Workshop on Human Behavior Understanding.

[29] Ji S., Xu W., Yang M., Yu K. (2012). 3D convolutional neural networks for human action recognition. IEEE Trans. Pattern Anal. Mach. Intell..

[30] Grushin A., Monner D., Reggia J., Mishra A. Robust human action recognition via long short-term memory. Proceedings of the 2013 International Joint Conference on Neural Networks (IJCNN).

[31] Sun L., Jia K., Yeung D., Shi B. Human action recognition using factorized spatio-temporal convolutional networks. Proceedings of the IEEE International Conference on Computer Vision.

[32] Simonyan K., Zisserman A. (2014). Two-stream convolutional networks for action recognition in videos. arXiv.

[33] Park E., Han X., Berg T., Berg A. Combining multiple sources of knowledge in deep cnns for action recognition. Proceedings of the 2016 IEEE Winter Conference on Applications of Computer Vision (WACV).

[34] Wang H., Schmid C. Action recognition with improved trajectories. Proceedings of the IEEE International Conference on Computer Vision.

[35] Peng X., Zou C., Qiao Y., Peng Q. (2014). Action recognition with stacked fisher vectors. European Conference on Computer Vision.

[36] Wang L., Qiao Y., Tang X. Action recognition with trajectory-pooled deep-convolutional descriptors. Proceedings of the IEEE Conference on Computer Vision and Pattern Recognition.

[37] Ahsan U., Sun C., Essa I. (2018). DiscrimNet: Semi-Supervised Action Recognition from Videos using Generative Adversarial Networks. arXiv.

[38] Shi L., Zhang Y., Cheng J., Lu H. Two-Stream Adaptive Graph Convolutional Networks for Skeleton-Based Action Recognition. Proceedings of the IEEE/CVF Conference on Computer Vision and Pattern Recognition (CVPR).

[39] Li M., Chen S., Chen X., Zhang Y., Wang Y., Tian Q. Actional-structural graph convolutional networks for skeleton-based action recognition. Proceedings of the IEEE/CVF Conference on Computer Vision and Pattern Recognition.

[40] Zhou Y., Sun X., Zha Z., Zeng W. Mict: Mixed 3d/2d convolutional tube for human action recognition. Proceedings of the IEEE Conference on Computer Vision and Pattern Recognition.

[41] Feichtenhofer C., Pinz A., Zisserman A. Convolutional two-stream network fusion for video action recognition. Proceedings of the IEEE Conference on Computer Vision and Pattern Recognition.

[42] Akilan T., Wu Q., Safaei A., Jiang W. A late fusion approach for harnessing multi-CNN model high-level features. Proceedings of the 2017 IEEE International Conference on Systems, Man, and Cybernetics (SMC).

[43] Foggia P., Saggese A., Strisciuglio N., Vento M. Exploiting the deep learning paradigm for recognizing human actions. Proceedings of the 2014 11th IEEE International Conference on Advanced Video and Signal Based Surveillance (AVSS).

[44] Chun S., Lee C. (2016). Human action recognition using histogram of motion intensity and direction from multiple views; Human action recognition using histogram of motion intensity and direction from multiple views. IET Comput. Vis..

[45] Baumann F., Ehlers A., Rosenhahn B., Liao J. (2016). Recognizing human actions using novel space-time volume binary patterns. Neurocomputing.

[46] Jiang Z., Lin Z., Davis L. (2012). Recognizing human actions by learning and matching shape-motion prototype trees. IEEE Trans. Pattern Anal. Mach. Intell..

[47] Tahir M., Bouridane A., Kurugollu F. (2007). Simultaneous feature selection and feature weighting using Hybrid Tabu Search/K-nearest neighbor classifier. Pattern Recognit. Lett..

[48] Villegas M., Paredes R. (2011). Dimensionality reduction by minimizing nearest-neighbor classification error. Pattern Recognit. Lett..

[49] Weinberger K., Blitzer J., Saul L. (2005). Distance metric learning for large margin nearest neighbor classification. Adv. Neural Inf. Process. Syst..

[50] Li W., Wong Y., Liu A., Li Y., Su Y., Kankanhalli M. Multi-camera action dataset for cross-camera action recognition benchmarking. Proceedings of the 2017 IEEE Winter Conference on Applications of Computer Vision (WACV).

[51] Weinl D., Boyer E., Ronfard R. Action recognition from arbitrary views using 3D exemplars. Proceedings of the 2007 IEEE 11th International Conference on Computer Vision.

[52] Faraki M., Palhang M.S., Erson C. (2015). Log-Euclidean bag of words for human action recognition. IET Comput. Vis..

[53] Ullah A., Muhammad K., Hussain T., Baik S. (2021). Conflux LSTMs network: A novel approach for multi-view action recognition. Neurocomputing.

[54] Naeem H.B., Murtaza F., Yousaf M., Velastin S. (2021). T-VLAD: Temporal vector of locally aggregated descriptor for multiview human action recognition. Pattern Recognit. Lett..

[55] Malik N., Abu Bakar S., Sheikh U. (2022). Multiview Human Action Recognition System Based on OpenPose and KNN Classifier. Proceedings of the 11th International Conference on Robotics, Vision, Signal Processing and Power Applications.

[56] Sargano A., Angelov P., Habib Z. (2016). Human Action Recognition from Multiple Views Based on View-Invariant Feature Descriptor Using Support Vector Machines. Appl. Sci..

[57] Vishwakarma D., Kapoor R., Dhiman A. (2016). A proposed unified framework for the recognition of human activity by exploiting the characteristics of action dynamics. Robot. Auton. Syst..

[58] Pehlivan S., Duygulu P. (2011). A new pose-based representation for recognizing actions from multiple cameras. Comput. Vis. Image Underst..

[59] Kiruba K., Shiloah E., Sunil R. (2019). Hexagonal volume local binary pattern (H-VLBP) with deep stacked autoencoder for human action recognition. Cogn. Syst. Res..

[60] Kiran S., Khan M., Javed M., Alhaisoni M., Tariq U., Nam Y., Damasevicius R., Sharif M. (2021). Multi-layered deep learning features fusion for human action recognition. Comput. Mater. Contin..

[61] Shahroudy A., Liu J., Ng T., Wang G. Ntu rgb+ d: A large scale dataset for 3d human activity analysis. Proceedings of the IEEE Conference on Computer Vision and Pattern Recognition.

[62] Rahmani H., Mian A. 3d action recognition from novel viewpoints. Proceedings of the IEEE Conference on Computer Vision and Pattern Recognition.

[63] Ke Q., Bennamoun M., An S., Sohel F., Boussaid F. A new representation of skeleton sequences for 3d action recognition. Proceedings of the IEEE Conference on Computer Vision and Pattern Recognition.

[64] Li S., Li W., Cook C., Zhu C., Gao Y. Independently recurrent neural network (indrnn): Building a longer and deeper rnn. Proceedings of the IEEE Conference on Computer Vision and Pattern Recognition.

[65] Yan S., Xiong Y., Lin D. Spatial temporal graph convolutional networks for skeleton-based action recognition. Proceedings of the Thirty-second AAAI Conference on Artificial Intelligence.

